# Identification of the Pyroptosis-Related Prognosis Gene Signature and Immune Infiltration in Hepatocellular Carcinoma

**DOI:** 10.1155/2022/9124216

**Published:** 2022-04-30

**Authors:** Heng Yu, Xue Bai, Wangyang Zheng

**Affiliations:** ^1^Department of Digestive Medicine, South China Hospital, Health Science Center, Shenzhen University, Shenzhen 518116, China; ^2^Department of Clinic of Internal Medicine, Ulm University, Ulm, Germany; ^3^Department II of Gastroenterology, Third Affiliated Hospital of Harbin Medical University, Harbin 150086, China

## Abstract

Hepatocellular carcinoma (HCC) is one of the most heterogeneous malignancies worldwide with a dismal prognosis. Lack of efficient biomarkers, early detection, and prognosis is still a challenge for HCC. Pyroptosis is a new discovery inflammatory form of programmed cell death. There is growing evidence revealed that pyroptosis plays a role in physiological and pathological conditions of human cancers. However, the prognostic evaluation of these pyroptosis-related genes (PRGs) in HCC remains blank. Consensus clustering of PRGs was used to classify 374 patients with HCC from the TCGA-LIHC cohort. By applying the least absolute shrinkage and selection operator (LASSO) Cox regression method, a 2-gene prognostic gene model (PLCG1 and GSDMC) was built and indicated the survival rate in HCC with medium-to-high accuracy. Then, the median risk score from the TCGA cohort was utilized; the prognostic gene model was also accurate in Gene Expression Omnibus (GEO) cohort. The functional enrichment analysis indicated that the oncogenic properties are associated with prominent hallmarks of cancer. The ssGSEA analyses and TIMER database indicated that immune infiltration tumor microenvironment in the HCC. In conclusion, our findings provide a foundation for further research targeting PRGs and their immune microenvironment.

## 1. Introduction

Hepatocellular carcinoma (HCC) ranks the sixth most lethal malignancy and accounts for the second leading cause of cancer-related deaths worldwide [1]. The development of HCC refers to multiply steps. The nonresolving inflammation is a significant driver of disease, causing the tumor often rise in inflammatory conditions such as hepatitis B virus (HBV) and hepatitis C virus (HCV) infection, liver cirrhosis, nonalcoholic fatty liver, or alcoholic liver [2].

Pyroptosis was first described as a novel type of cell suicide in macrophages, which are infected by Shigella flexneri [3]. Not until 2001, the word “pyroptosis” was defined to distinguish it from apoptosis. Further research found pyroptosis was the two-sided sword for cell survival. Moderate pyroptosis improves immune activity and helps protect these pathogens [4]. The excessive one may construct an unfavorable inflammatory immune microenvironment, which may fasten diseases progressing, especially in cancer pathology [5]. Although studies of pyroptosis in HCC are just in their infancy, there are also many researches in this area [6, 7]. These studies highlight the vital role of pyroptosis in HCC. Moreover, pyroptosis is also found to affect the immune activity of HCC [8]. However, whether these pyroptosis-related genes are correlated with HCC patient prognosis and reflection in immune activity remain largely unknown. The new genome sequencing technique and the public databases allow us to explore the adequate sample size and available multiomics data systematically. The ferroptosis-related genes also showed great power in HCC survival prediction [9]. In the present study, we identified pyroptosis-related gene expressions, established a pyroptosis prognostic gene model with survival prediction, and evaluated the model in HCC patients. Moreover, combined with clinicopathological features of patients, a nomogram-based risk assessment of patients was constructed to improve the prediction ability and accuracy of the model. Finally, GSEA, ssGSEA, and TIMER databases were employed to assess their underlying mechanical pathways and immune cell infiltration and activity in the tumor microenvironment (TME).

## 2. Materials and Methods

### 2.1. HCC Datasets and Preprocessing

We obtained level 3 RNA sequencing (RNA-Seq) data and clinic information of the HCC cohort downloaded from the TCGA database (https://cancergenome.nih.gov). This cohort contained 374 HCC tumor tissues and 50 normal tissues with gene expression profiles, which was used in the train set. The test group's RNA-Seq data and clinical information for external validation were downloaded from the GEO database (https://www.ncbi.nlm.nih.gov/geo/, ID: GSE54236). Data analysis was performed with the R (version 3.6.1) and R Bioconductor packages. We use the STRING database to construct performing the interaction of protein interaction [10].

### 2.2. Identification and Validation of the Prognostic Pyroptosis-Related Gene Signature

We obtained 33 PRGs from prior reviews [11–16] and the MSigDB database. They are shown in Table S1. We employed the univariate and multivariate Cox regression analyses to evaluate each gene impact on patients' survival status in TCGA cohort Only PEGs with a *P* value < 0.05 and logFC > 1.5 were used for further study. Then, the “limma” package was used to identify the total differentially expressed genes (DEGs) between tumor and normal tissues with a *P* value < 0.05 in genes in HCC. The intersect genes of two sets were used to construct the gene prognosis model. The LASSO analysis with ten cross-validations was applied using the “glmnet” R package. According to the best lambda value, only two PRG prognostic genes list with coefficients were generated from the LASSO model. Each patient's risk score can be obtained from the gene expression level and corresponding coefficients. The formula is calculated as follows: score = expression gene one∗coefficient + expression gene two∗coefficient and so on. Based on the median value of the risk score, the patients were classified into the high risk or low-risk groups. Then predictive accuracy of the model was evaluated by time receiver-operating characteristic (ROC) analysis. We also perform the Kaplan–Meier survival analysis, PCA, and t-SNE to visualization this model by R packages. Using the cut-off value from TCGA, we evaluated the accuracy of this model in the GEO group. After external evaluation, combined with these clinicopathologic features, we constructed the nomogram to predict the survival probability of HCC patients. The calibration curves were used to assess the accuracy of this nomogram.

### 2.3. Functional Enrichment Analysis

The “clusterProfiler” R package was used in the GO enrichment analysis. We employed GSEA (gene set enrichment analysis) in the JAVA environment to assess the possible mechanisms between the high- and low-risk groups. The entire dysregulated genes between tumor and normal samples were used for GSEA. The random sample permutations number was set at 1000. And *P* value < 0.05 and FDR *q* value < 0.05 were set as the significance.

### 2.4. TME Immune Cell Infiltration

We employed an online TIMER database to comprehensively analyze these PRG prognostic genes' effect on tumor-infiltrating immune cells (https://cistrome.shinyapps.io/timer/) [17]. The ssGSEA (single sample GSEA) was also used in this section.

### 2.5. Statistical Analysis

The data was conducted using the R software (3.6.1). Moreover, the PEG expression levels between the tumor and normal tissues were compared with one-way ANOVA. The log-rank test was used in the comparison of Kaplan–Meier plots. *P* < 0.05 was deemed statistically significant.

## 3. Results

### 3.1. Identification of Prognostic Pyroptosis-Related DEGs

**Figure 1:**
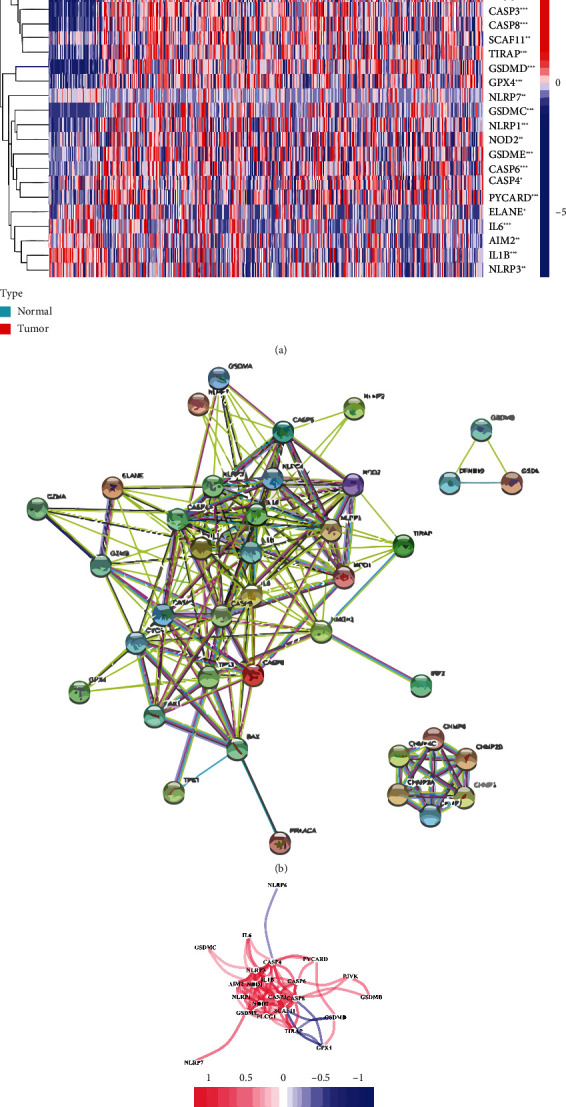
Identification of the candidate PRGs in the TCGA cohort. (a) The heat map for different expressions of PRGs between HCC and normal tissues (green: low expression; red: high expression). (b) The PPI network from the STRING database indicated the interactions among the PRGs. (c) The correlation network of PRGs.

**Figure 2:**
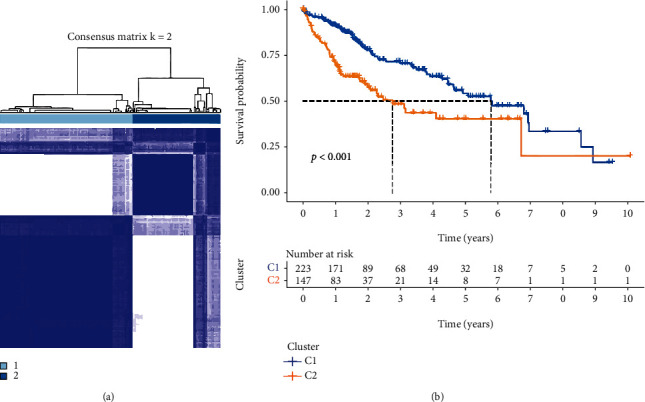
Pyroptosis-related regulators relate to subgroups of HCC. (a) Two clusters were likely to be grouped based on the expression of 33 PRGs. (b) The OS curves for the two clusters.

A total of 33 were included in our study (Table S1). Most PRGs (26/33) were differentially expressed between tumor and normal tissues (Figure 1(a)) (all FDR < 0.05). Among these ELANE, NLRP6, CASP4, SCAF11, PRKACA, CASP6, CASP9, AIM2, NLRP7, GPX4, NOD2, TIRAP, PJVK, CASP3, NOD1, CASP8, GSDMD, NLRP1, GSDME, GSDMB, PYCARD, PLCG1, and GSDMC were upregulated in tumor than normal, while IL6, IL1B, and NLRP3 were downregulated (Figure 1). The STRING database showed the protein-protein interaction (PPI) of these PRGs (Figure 1(b)). The correlation gene network containing all pyroptosis-related genes is presented in Figure 1(c). Based on these dysregulated genes, we identified two different regulation patterns in the TCGA cohort (Figure 2(a)). The survival analysis showed that many advantages of cluster 1 were higher than that of cluster 2 (Figure 2(b)). To focus on the most dysregulated PRGs between two groups, we took these genes (PLCG1, GSDMC, PYCARD) whose logFC is greater than 1.5 into the prognostic model. Then, the R package “limma” was used to screen all kinds of dysregulated genes between tumor and normal. The lap of two gene sets was used in our prognostic gene mode (PLCG1, GSDMC).

### 3.2. Construction of a Prognostic Model in the TCGA

**Figure 3:**
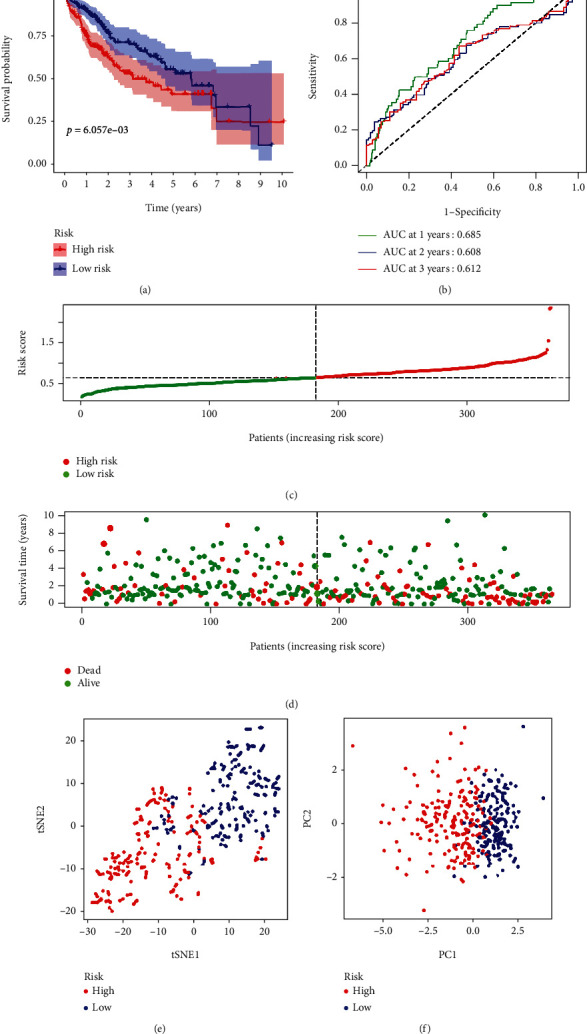
Prognostic evaluation of the signature model in the TCGA cohort. (a) Kaplan-Meier curves for the OS of HCC patients in the high-risk group and low-risk group. (b) The time-dependent ROC curves verified the predictive performance of the risk score. (c) The distribution of the risk scores in the TCGA cohort. (d) The distributions of OS status, OS, and risk score for TCGA patients. (e) The t-SNE analysis of the TCGA cohort. (f) The PCA plot of the TCGA cohort.

LASSO Cox regression analysis was employed to establish a prognostic model using the expression profile of the two genes above. After identifying the optimal value of *λ*, the risk score was calculated as follows: 0.24850607345869∗ expression level of PLCG1+0.418060351852712∗ expression level of GSDMC. According to the median cut-off value, the patients were stratified into high-risk or low-risk groups. The Kaplan-Meier analysis indicated that the high-risk group patients had a significantly worse OS than their low-risk counterparts (Figure 3(a)). Time-dependent ROC curve for OS was employed to evaluate the predictive performance of the risk score. The area under the curve (AUC) reached 0.685 at 1 year, 0.608 at 2 years, and 0.612 at 3 years (Figure 3(b)). PCA (principal component analysis) and t-SNE analysis also indicated that different risk group patients were distributed in two directions (Figures 3(e) and 3(f)).

### 3.3. External Validation of the Risk Signature

**Figure 4:**
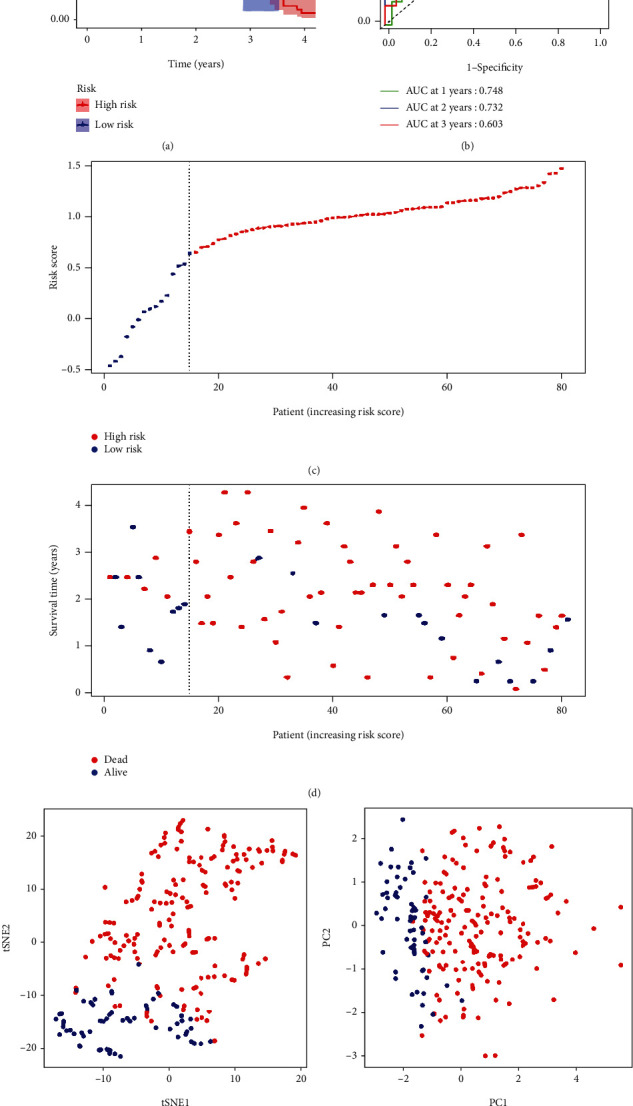
Validation of the prognosis signature in the GEO cohort. (a) Kaplan-Meier curves for the OS of HCC patients in the high-risk and low-risk groups. (b) The time-dependent ROC curves verified the prognostic performance of the risk score. (c) The distribution of the risk scores in the GEO cohort. (d) The distributions of OS status, OS, and risk score in the GEO cohort. (e) The t-SNE analysis of the GEO cohort. (f) The PCA plot of the GEO cohort.

82 HCC patients from Gene Expression Omnibus (GEO) cohort (GSE54236) were utilized in the validation set. Based on the TCGA median risk score, patients were divided into high or low-risk groups. Kaplan–Meier plot indicated a significant OS difference in the survival rate between the two groups (Figure 4(a)). The AUC of the ROC curve showed good predictive efficacy (AUC = 0.748 for 1-year, 0.732 for 2-year, and 0.603 for 3-year survival) (Figure 4(b)). The PCA and tSNE1 also showed great separation between tumor and normal (Figures 4(e) and 4(f)).

### 3.4. Independent Prognostic Value of the Risk Model

**Figure 5:**
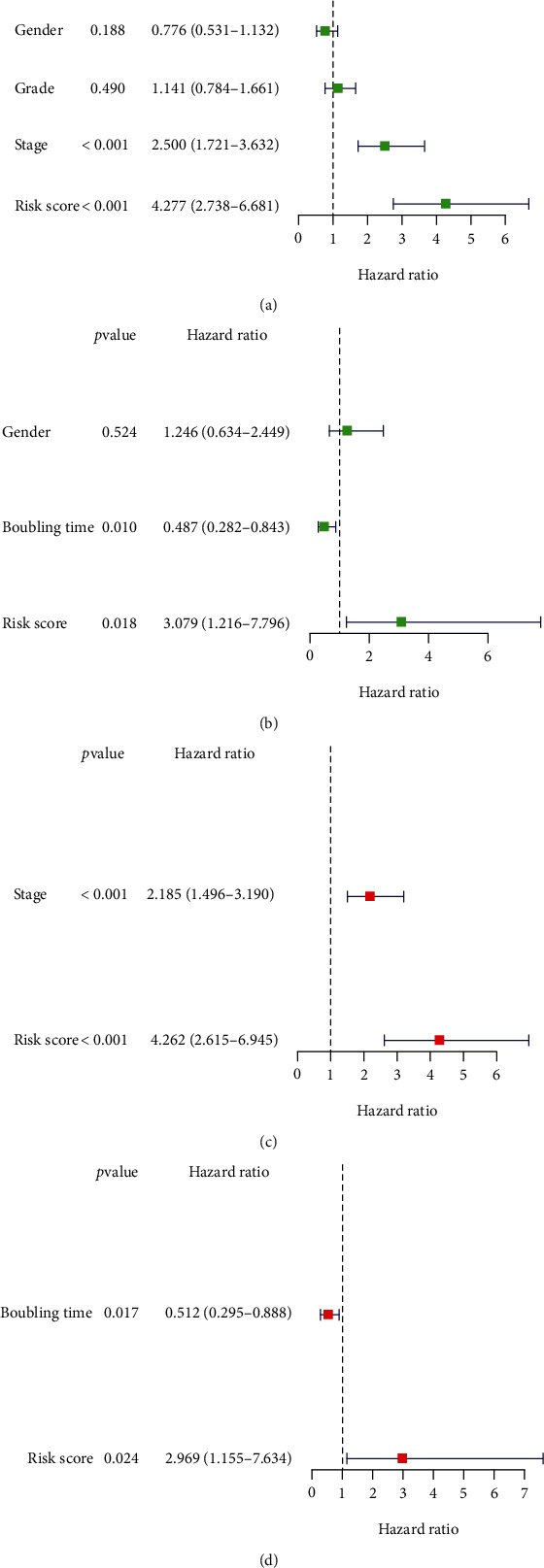
Univariate and multivariate Cox regression analyses for the risk score. (a) The univariate Cox regression result for TCGA. (b) The multivariate Cox regression result for TCGA. (c) The univariate Cox regression result for GEO. (d) The multivariate Cox regression result for GEO.

Univariate and multivariable Cox regression analyses were also used to evaluate whether the risk score is an independent prognostic factor for HCC survival. The result of the univariate Cox regression analysis indicated that the risk score was an independent factor predicting poor survival in both the TCGA and GEO (HR = 4.277, 95% CI: 2.738–6.681 and HR: 3.079, 95% CI: 1.216–7.796, Figures 5(a) and 5(b)). The multivariate analysis also implied that the risk score as a prognostic factor (HR = 4.262, 95% CI: 2.615–6.945 and HR: 2.969, 95% CI: 1.155–7.634, Figures 5(c) and 5(d)).

### 3.5. Building a Predictive Nomogram and the Calibration Curves

**Figure 6:**
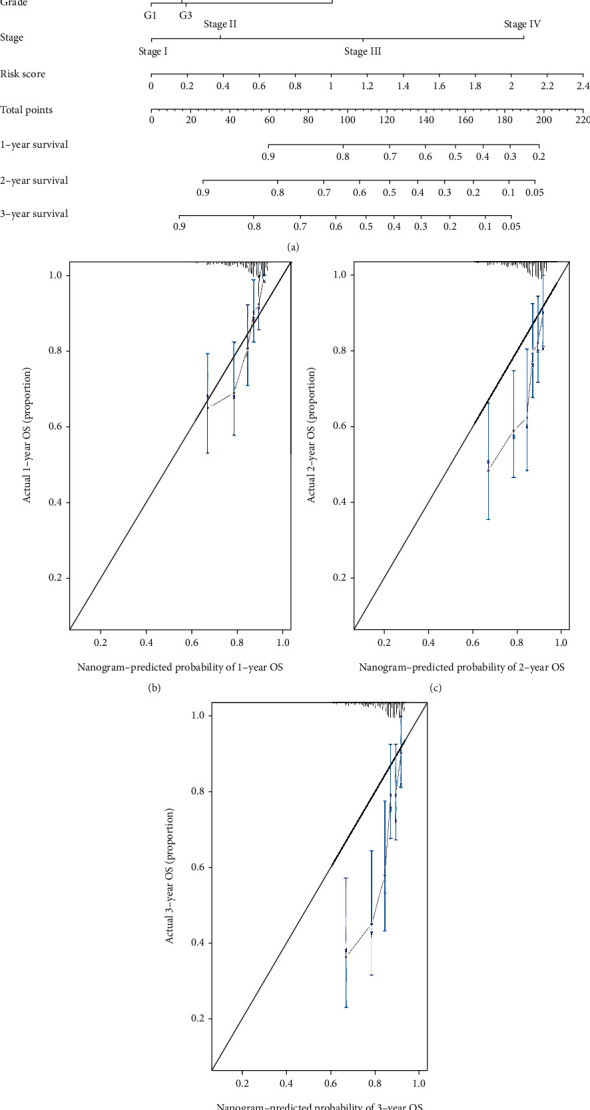
Construction of a predictive nomogram. (a) Nomogram to predict the 1-year, 2-year, and 3-year overall survival rate of HCC patients. (b) The calibration curve for the 1-year survival for the TCGA. (c) The calibration curve for the 2-year survival for the TCGA. (d) The calibration curve for the 3-year survival for the TCGA.

Combined with these clinicopathologic features, the risks core was used to build a predictive nomogram to predict the survival probability (Figure 6(a)). The c-index of the nomogram was 0.68. The calibration curves were employed to assess the accuracy of this nomogram (Figures 6(b)–6(d)).

### 3.6. Functional Analyses, TME Immune Cell Infiltration, and Functions

**Figure 7:**
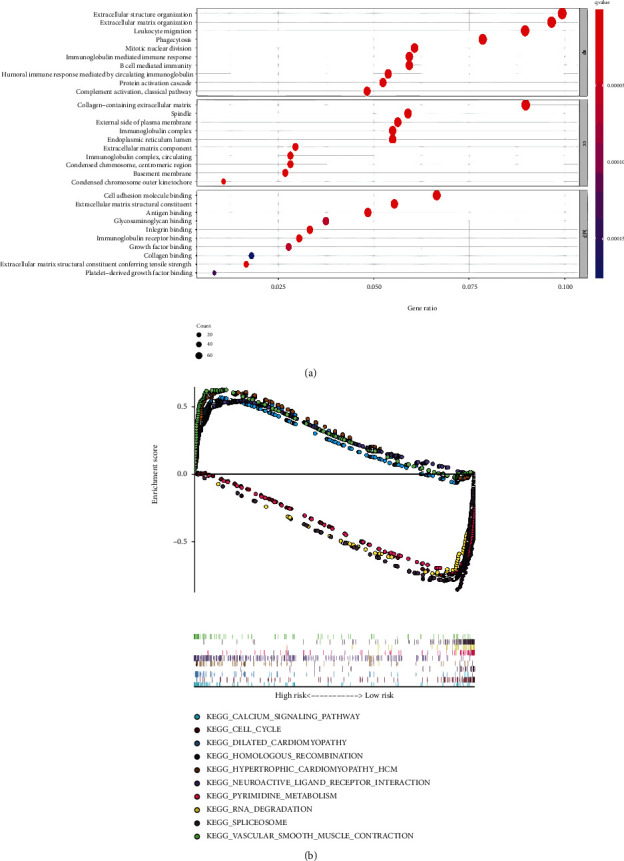
The functional analysis of DEGs between the high- and low-risk groups in the TCGA. (a) Bubble graph for GO enrichment. (b) GSEA analysis for KEGG pathway.

To elucidate the biological behavior and pathways associated with the risk score, we perform the GO analysis and GSVA enrichment analysis using the DEGs between the high-risk and low-risk groups (Figures 7(a) and 7(b)). As shown in Figure 7(a), the DEGs were associated with many immune response pathways and many extracellular structure organization pathways. The KEGG pathway analysis showed that DEGs were rich in cell cycle, RNA degradation pathways, etc.

**Figure 8:**
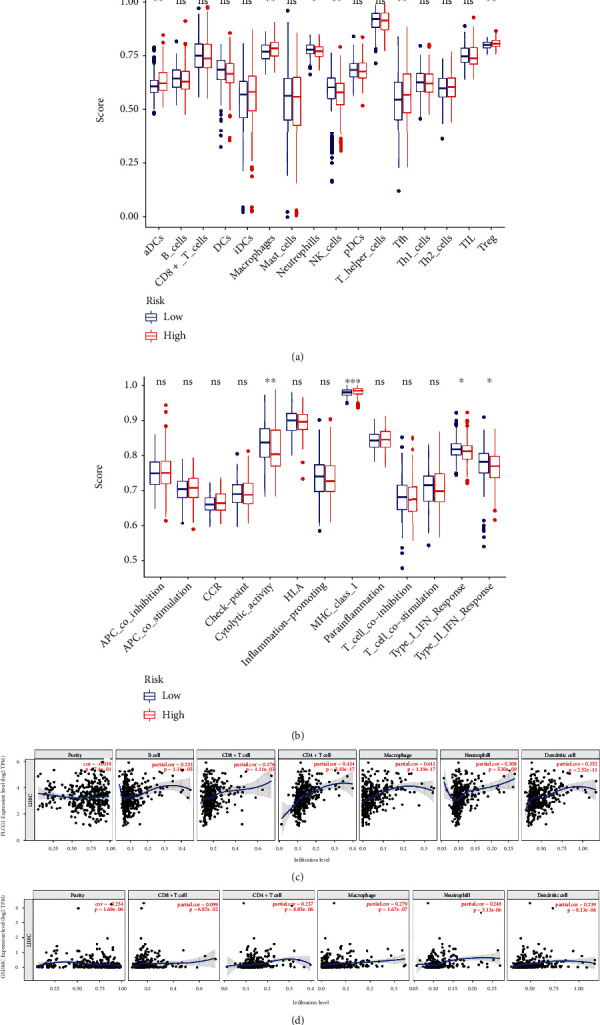
The association of two PRGs and immune activities. (a) The ssGSEA scores for TME immune cell infiltration. (b) The ssGSEA scores for TME immune cell functions. (c) The correlation coefficients of PLCG1 and immune cells (TIMER database). (d) The correlation coefficients of GSDMC and immune cells (TIMER database).

Therefore, ssGSEA (single-sample gene set enrichment analysis) was used to compare the 16 types of immune cells and their activity and TME functions. In the TCGA cohort, the high-risk subgroup had a higher rate of aDCs, Tfh, and Treg cells while lower macrophage cells, neutrophils, and Nk cells rate (Figure 8(a)). Moreover, the cytolytic activity and type 1 and type 2 IFN responses were lower in the high-risk group than low-risk, while the MHC class 1 activity was greater (Figure 8(b)). Then, we used the TIMER database to analyze the correlation of expression of two prognostic PRGs and immune infiltration in HCC. As shown in Figure 7(c), PLCG1 expression was positive with the immune infiltration level of CD4+, macrophages, neutrophils, and dendritic cells. GSDMC expression was positive with CD8+, CD4+, macrophages, neutrophil, and dendritic cells, while negative with purity.

## 4. Discussion

Chronic infection such as virus infection, liver cirrhosis, nonalcoholic fatty liver, or alcoholic liver leads to gradual development of HCC progression. As the newly defined inflammation-associated programmed cell death, pyroptosis has been proved to have actual effects on cancers. Its two side effects are always upon the tumor microenvironments. On the one hand, normal cells could be provoked by a large number of pyroptosis derived inflammatory factors, which leads to transformation into tumor cells [18]. On the other hand, promoting cell pyroptosis could kill these tumor cells, release the tumor burden, and cure diseases. Some studies have already identified the antitumor effect of pyroptosis in HCC and colorectal cancer [6, 7, 19]. Moreover, the pyroptosis phenomena have been proven to connect with TME immune activity [7]. Studies have identified that molecular subgroup classification was associated with distinct clinical outcomes in solid tumors [15, 20]. Therefore, we need to explore the changes in the different status and mechanisms of HCC associated with the pyroptosis and immune environment to facilitate treatment. So, employing all the genes related to pyroptosis, we explored a prognostic signature for HCC patients. Our model provides PLCG1, and GSDMC could be biomarkers and potential targets for antitumor therapy and impact the HCC immune microenvironment.

Phospholipase C gamma 1 (PLCG1) participates in receptor tyrosine kinase- (RTK-) mediated signal transduction pathway. Knockdown of PLCG1 may interrupt the GSDMD mediated pyroptosis [21]. The PLCG1 expression could also be a biomarker for myelodysplastic syndromes and oral squamous cell carcinoma [22, 23]. The dysregulated PLCG1 in HCC tissues and cell lines was also proved [8]. PLCG1 was also taking part in lung cancer pathology [24]. In our prognosis model, high PLCG1 expression was also correlated with poor survival outcomes, which may result from the low activity of pyroptosis. GSDMC, also called melanoma-derived leucine zipper extranuclear factor (MLZE), was first described from melanoma cells. Its expression was higher in the gastrointestinal tract and skin than in other normal tissues [25]. Moreover, GSDMC functions as an oncogene in multiply cancers and may serve as a potential therapeutic target. GSDMC stimulated colorectal cancer cell proliferation by interacting with transforming growth factor *β* receptor type II [26]. Along with PD-L1, GSDMC leads to tumor's necrosis [27]. Upregulated GSDMC also refers to poor clinical outcomes for lung cancer, breast cancer, and melanoma [27–29], similar to our findings in HCC.

Recently, some papers have focused on the prognostic significance of PRGs in HCC. Liu et al. built a prognostic model based on CASP1, CHMP6, CASP4, DHX9, GZMA, and DFNA5 expression [30]. Chen et al. screened six genes in the model: BAK1, CHMP4B, DHX9, GSDMC, GSDME, and TREM2 expressions [31]. Fu and Song constructed a model with GSDME, GPX4, and SCAF11 [32]. Chen et al. build with BAK1, CHMP4B, DHX9, GSDMC, GSDME, and TREM2 expressions [33]. In our opinion, the more genes used in the prognostic model, the more this model will cost, and the less possible it will be used in the clinic. So, we only choose two genes in our model. Although the underlying mechanisms of pyroptosis are still covered, our study may help doctors classify HCC patients into subtypes, build a pyroptosis-related prognostic model, and then externally validate. Furthermore, functional enrichment and immune impact were also performed. There are still some limitations that are needed to be considered. All data we used were from public databases. More individual clinical information is needed to improve our model. As we only consider these hallmark genes to build a prognostic model, these less prominent genes may also significantly affect tumor progression. More in vitro and in vivo experiments are required for studying these underlying mechanisms behind the phenomena. Tumor heterogeneity is also substantial in tumor microenvironment studies.

In summary, our study demonstrated that pyroptosis is closely connected to HCC progression. We provided a novel pyroptosis-related gene signature to predict patients' survival. With further verification, it is believed that this model could be successfully used in clinic.

## Data Availability

The datasets generated for this study can be found in the TCGA and GEO-GSE54236. Further inquiries can be directed to the corresponding authors.

## Supplementary Materials

Supplementary Materials Table S1: the 33 pyroptosis-related genes from prior reviews and MSigDB database.

## References

[1] Ferlay J., Soerjomataram I., Dikshit R. (2015). Cancer incidence and mortality worldwide: sources, methods and major patterns in GLOBOCAN 2012. *International Journal of Cancer*.

[2] Bertino G., Demma S., Ardiri A. (2014). Hepatocellular carcinoma: novel molecular targets in carcinogenesis for future therapies. *BioMed Research International*.

[3] Zychlinsky A., Prevost M. C., Sansonetti P. J. (1992). Shigella flexneri induces apoptosis in infected macrophages. *Nature*.

[4] Cookson B. T., Brennan M. A. (2001). Pro-inflammatory programmed cell death. *Trends in Microbiology*.

[5] Ahechu P., Zozaya G., Martí P. (2018). NLRP3 inflammasome: a possible link between obesity-associated low-grade chronic inflammation and colorectal cancer development. *Frontiers in Immunology*.

[6] Wei Q., Zhu R., Zhu J., Zhao R., Li M. (2019). E2-induced activation of the NLRP3 inflammasome triggers pyroptosis and inhibits autophagy in HCC cells. *Oncology Research*.

[7] Chen Z., He M., Chen J., Li C., Zhang Q. (2020). Long non-coding RNA SNHG7 inhibits NLRP3-dependent pyroptosis by targeting the miR-34a/SIRT1 axis in liver cancer. *Oncology Letters*.

[8] Hage C., Hoves S., Strauss L. (2019). Sorafenib induces pyroptosis in macrophages and triggers natural killer cell–mediated cytotoxicity against hepatocellular carcinoma. *Hepatology*.

[9] Liang J. Y., Wang D. S., Lin H. C. (2020). A novel ferroptosis-related gene signature for overall survival prediction in patients with hepatocellular carcinoma. *International Journal of Biological Sciences*.

[10] Szklarczyk D., Franceschini A., Kuhn M. (2011). The STRING database in 2011: functional interaction networks of proteins, globally integrated and scored. *Nucleic Acids Research*.

[11] Shi J., Zhao Y., Wang K. (2015). Cleavage of GSDMD by inflammatory caspases determines pyroptotic cell death. *Nature*.

[12] He W. T., Wan H., Hu L. (2015). Gasdermin D is an executor of pyroptosis and required for interleukin-1*β* secretion. *Cell Research*.

[13] Orning P., Weng D., Starheim K. (2018). Pathogen blockade of TAK1 triggers caspase-8-dependent cleavage of gasdermin D and cell death. *Science*.

[14] Zhou Z., He H., Wang K. (2020). Granzyme A from cytotoxic lymphocytes cleaves GSDMB to trigger pyroptosis in target cells. *Science*.

[15] Zhang Z., Zhang Y., Xia S. (2020). Gasdermin E suppresses tumour growth by activating anti-tumour immunity. *Nature*.

[16] Shao W., Yang Z., Fu Y. (2021). The pyroptosis-related signature predicts prognosis and indicates immune microenvironment infiltration in gastric cancer. *Frontiers in Cell and Development Biology*.

[17] Li B., Severson E., Pignon J. C. (2016). Comprehensive analyses of tumor immunity: implications for cancer immunotherapy. *Genome Biology*.

[18] Karki R., Kanneganti T. D. (2019). Diverging inflammasome signals in tumorigenesis and potential targeting. *Nature Reviews. Cancer*.

[19] Zaki M. H., Vogel P., Body-Malapel M., Lamkanfi M., Kanneganti T. D. (2010). IL-18 production downstream of the Nlrp3 inflammasome confers protection against colorectal tumor formation. *Journal of Immunology*.

[20] Roepman P., Schlicker A., Tabernero J. (2014). Colorectal cancer intrinsic subtypes predict chemotherapy benefit, deficient mismatch repair and epithelial-to-mesenchymal transition. *International Journal of Cancer*.

[21] Kang R., Zeng L., Zhu S. (2018). Lipid peroxidation drives gasdermin D-mediated pyroptosis in lethal polymicrobial sepsis. *Cell Host & Microbe*.

[22] Shiseki M., Ishii M., Miyazaki M. (2020). Reduced PLCG1 expression is associated with inferior survival for myelodysplastic syndromes. *Cancer Medicine*.

[23] Zhu D., Tan Y., Yang X. (2014). Phospholipase C gamma 1 is a potential prognostic biomarker for patients with locally advanced and resectable oral squamous cell carcinoma. *International Journal of Oral and Maxillofacial Surgery*.

[24] Song W., Kim L. C., Han W. (2020). Phosphorylation of PLC*γ*1 by EphA2 receptor tyrosine kinase promotes tumor growth in lung cancer. *Molecular Cancer Research*.

[25] Tamura M., Tanaka S., Fujii T. (2007). Members of a novel gene family, Gsdm, are expressed exclusively in the epithelium of the skin and gastrointestinal tract in a highly tissue-specific manner. *Genomics*.

[26] Miguchi M., Hinoi T., Shimomura M. (2016). Gasdermin C is upregulated by inactivation of transforming growth factor *β* receptor type II in the presence of mutated Apc, promoting colorectal cancer proliferation. *PLoS One*.

[27] Hou J., Zhao R., Xia W. (2020). PD-L1-mediated gasdermin C expression switches apoptosis to pyroptosis in cancer cells and facilitates tumour necrosis. *Nature Cell Biology*.

[28] Shou Y., Yang L., Yang Y., Zhu X., Li F., Xu J. (2020). Identification of signatures of prognosis prediction for melanoma using a hypoxia score. *Frontiers in Genetics*.

[29] Wei J., Xu Z., Chen X. (2020). Overexpression of GSDMC is a prognostic factor for predicting a poor outcome in lung adenocarcinoma. *Molecular Medicine Reports*.

[30] Liu S., Shao R., Bu X., Xu Y., Shi M. (2021). Identification of the pyroptosis-related gene signature for overall survival prediction in patients with hepatocellular carcinoma. *Frontiers in cell and developmental biology*.

[31] Chen J., Tao Q., Lang Z. (2022). Signature construction and molecular subtype identification based on pyroptosis-related genes for better prediction of prognosis in hepatocellular carcinoma. *Oxidative Medicine and Cellular Longevity*.

[32] Fu X., Song C. (2021). Identification and validation of pyroptosis-related gene signature to predict prognosis and reveal immune infiltration in hepatocellular carcinoma. *Frontiers in Cell and Development Biology*.

[33] Chen Z., Zou Y., Zhang Y. (2022). A pyroptosis-based prognostic model for immune microenvironment estimation of hepatocellular carcinoma. *Disease Markers*.

