# Supplementary material for: Identification of the Pyroptosis-Related Prognosis Gene Signature and Immune Infiltration in Hepatocellular Carcinoma
Source: Dis Markers. 2022 Apr 30;2022:9124216. doi: 10.1155/2022/9124216 (PMC9078841; doi:10.1155/2022/9124216)
Supplement: Supplementary Materials — Table S1: the 33 pyroptosis-related genes from prior reviews and MSigDB database. [file 9124216.f1.doc]

Table S1. The 33 pyroptosis-related gene from prior reviews and MSigDB database.

| Genes |
| --- |
| PRKACA  GSDMB  SCAF11  PJVK  CASP9  NOD1  PLCG1  NLRP1  GSDME  ELANE  TIRAP  CASP4  GSDMD  GPX4  NLRP7  NLRP2  CASP3  CASP6  TNF  IL1B  IL18  CASP8  NLRP6  GSDMA  GSDMC  PYCARD  CASP5  AIM2  NOD2  NLRC4  NLRP3  IL6  CASP1 |
